# Hybrid Degradation Equipment Remaining Useful Life Prediction Oriented Parallel Simulation considering Model Soft Switch

**DOI:** 10.1155/2019/9179870

**Published:** 2019-03-12

**Authors:** Chenglong Ge, Yuanchang Zhu, Yanqiang Di

**Affiliations:** Shijiazhuang Campus, Army Engineering University, Shijiazhuang 050003, China

## Abstract

Equipment parallel simulation is an emerging simulation technology in recent years, and equipment remaining useful life (RUL) prediction oriented parallel simulation is an important branch of parallel simulation. An important concept in equipment parallel simulation is the model evolution driven by real-time data, including model selection and model parameter evolution. The current research on equipment RUL prediction oriented parallel simulation mainly focuses on a single continuous degradation mode, such as linear degradation and nonlinear degradation. Under this degradation condition, the model parameter evolution methods in parallel simulation can effectively predict equipment RUL. However, in practice, most of the equipment degradation processes exhibit a mixture of continuous degradation and discrete shock. So this requires adaptive selection of simulation models based on real-time degradation data. In this paper, the hybrid degradation equipment RUL prediction oriented parallel simulation considering model soft switch is studied. Firstly, under the modeling framework of the state space model (SSM), two kinds of degradation simulation models are established using the Wiener process and Poisson effect. Driven by the real-time degradation data, the model probability is calculated by using the forward interactive multiple model filtering algorithm to realize the model soft switch and data assimilation. On the basis of model soft switch, the expectation maximization algorithm is utilized to achieve model parameter evolution. Through the iteration between model soft switch and model parameter evolution, the simulation fidelity can be effectively improved and the actual equipment degradation state is continuously approached. According to the full probability theorem and the concept of first hitting time, the simulated degradation state distribution is integrated into the inverse Gaussian distribution. Then the analytical expression of the RUL probability density function is obtained to achieve RUL real-time prediction. Finally, a case study was conducted by using a bearing degradation data. The results show that the parallel simulation can effectively model the hybrid degradation process of the bearing. Compared with the single-model method that only considers the model parameter evolution, the RUL obtained by the method proposed in this paper has higher prediction accuracy and smaller uncertainty.

## 1. Introduction

As the equipment complexity increases and the time-varying property of the equipment-operating environment enhances, equipment maintenance has become increasingly complicated. Due to external shock, wear, fatigue, corrosion, and other reasons, the equipment's performance will be inevitably degraded, eventually causing equipment failure or even causing serious accidents. If we can determine the best opportunity for equipment maintenance and formulate the corresponding spare parts management and ordering plan based on the predicted RUL in the initial degradation stage, the reliability of equipment will be improved and the operational risks and operating costs of the equipment will be reduced effectively. In recent years, the prognostic and health management (PHM) technology has received more and more attention and has become an active investigation area in the reliability field [1]. PHM aims to accurately predict the equipment RUL. Accordingly, the reasonable maintenance and management of equipment are executed to guarantee the safety, reliability of equipment operation. The main tasks of PHM include RUL prediction and health management. Pecht and Chinnam [2, 3] both believe that RUL prediction is the core content of PHM and the RUL prognostic results provides a scientific basis for maintenance activities such as maintenance replacement and spare parts ordering. RUL prediction includes the probability density function (PDF) of RUL and its mathematical expectation. Since the RUL probability density function characterizes the uncertainty of RUL prediction, the probability density function is the primary predictor [4]. The current RUL prediction approaches can be broadly classified as failure physical model methods, statistics-based methods, and artificial intelligence methods [5]. For complex equipment, its failure mechanism is difficult to obtain, so the latter two methods get more attention. Statistics-based methods achieve RUL prognostic results via data fitting on the basis of statistics models. The commonly used statistics-based methods include the Markov chain [6], Bayesian method [7], inverse Gaussian process [8], Gamma process [9], Wiener process [10, 11], etc. The artificial intelligence methods achieve RUL prognostic results via data fitting mainly including machine learning. The commonly used artificial intelligence methods include neural network [12], support vector machine [13], etc. However, the artificial intelligence methods are limited by the two deficiencies in the practical application. One is that it usually cannot achieve the analytical expression of the probability density function of RUL, which restricts the real-time application of the methods [14]. The other is that it also requires a large amount of training samples, which usually cannot be satisfied in practice [15]. Contrastively, statistics-based methods do not depend on a large amount of training samples and are more flexible because of its independency regarding specific application objects, making it widely utilized in degradation modeling and RUL prediction. Among these statistics-based methods, the Wiener process is the most popular method because of the following advantages. On the one hand, compared with other methods (e.g., inverse Gaussian process and Gamma process), the Wiener process can model not only monotonous degradation paths but also nonmonotonous degradation paths. On the other hand, the Wiener process has excellent physical interpretation and mathematical property (i.e., its first hitting time distribution is inverse Gaussian distribution), which is helpful to obtain the analytical expression of the PDF of RUL.

Recent advances about Wiener process-based RUL prediction methods were discussed in the most recent review paper [16]. In this review, Zhang et al. systematically reviewed the conventional Wiener process-based RUL prediction methods and many generalizations and variants from the conventional Wiener process-based RUL prediction methods by considering the factors of nonlinearity [17], multisource variability [18], covariates [19], and multivariates [20]. In the above numerous applications, Wiener process-based methods showed a flexible modeling capability and has undergone extensive development. But there still exists a typical issue deserving further research. The issue is that it is usually assumed that the equipment degradation mode is a fixed continuous degradation in the entire degradation period among the Wiener process-based prediction methods. As a result, a single and fixed model is used for RUL prediction. However, besides the continuous degradation, the degradation mode may present a hybrid of multiple degradation modes in practice, which requires multiple models. Specially, the damage caused by the random shock is also an important reason accounting for equipment failure. In this paper, the degradation mode incorporating the continuous degradation and the random shock is called the hybrid degradation mode. Until now, in order to solve the RUL prediction issue of the hybrid degradation equipment, several researchers have attempted to establish the prognostic models on the basis of the Wiener process.

Si et al. [21] presented a new prognostic model to characterize the hybrid degradation equipment. In the proposed model, the linear Wiener model is used to describe the continuous degradation process and a compound Poisson process is utilized to characterize the randomly arriving shock. And each randomly arriving shock is described by a random variable which obeys the normal distribution with two parameters. Inspired by the research of Si et al. [21], Zhang et al. [22] regarded the hybrid degradation process as the state switching process and utilized a nonlinear Wiener process with state switching depicted by a continuous time Markov chain (CTMC) to describe the continuous degradation process. And also a random variable which obeys the normal distribution is used to characterize each shock introduced in the state switching. Regrettably, the model parameters estimation method was not investigated. On the basis of the previous studies [21, 22], Zhang et al. [23] proposed a hybrid degradation model with a continuous model described by a nonlinear Wiener process and a randomly arriving shock model characterized by a nonhomogeneous compound Poisson process. They not only obtained the approximated analytical lifetime under the concept of FHT, but also obtained parameters updating formulas by combining the expectation conditional maximization (ECM) algorithm and maximum likelihood estimation (MLE). Additionally, a numerical example and a case study of furnace wall were studied. Furthermore, similar to the previous study [23], Zhang et al. [24] utilized a specific nonlinear Wiener process with the power low model to describe the continuous degradation and the same method to describe the randomly arriving shock. On the basis of the above modeling mechanism, Du et al. [25] proposed a more generalized hybrid degradation model consisting of trend term and stochastic fluctuating term. It is worth noting that all the above latest research studies use one model to model the hybrid degradation process. Considering the unknown characteristic of arriving time and the amplitudes for the shock, it is a reasonable choice to construct multiple models to describe the hybrid degradation process and replace the model dynamically through model soft switch. Therefore, in order to accurately predict the RUL of hybrid degradation equipment, a novel real-time prediction method is needed urgently considering both the model soft switch and online evolution of model parameters. Equipment parallel simulation has become a possible solution [26, 27]. This method is called hybrid degradation equipment RUL prediction oriented parallel simulation.

**Figure 1:**
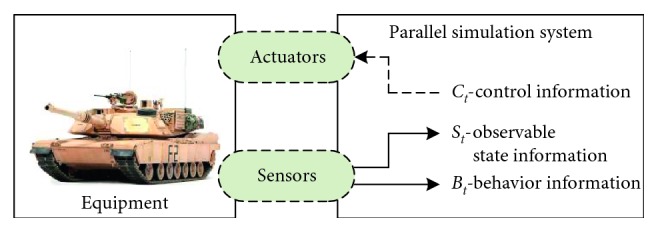
Overview of the equipment parallel simulation.

Equipment parallel simulation is an emerging simulation paradigm which aims to combine the simulation system with the actual equipment. It was originally defined at the AsiaSim/SCS AutumnSim conference in 2016. The simulation system benefits from the online acquisition of equipment information to update the simulation model. Conversely, the equipment benefits from the simulation results of the simulation system to improve the equipment performance. Particularly, the simulation system running in this mode is called the parallel simulation system. The equipment parallel simulation diagram is shown in Figure 1. In the equipment parallel simulation, the actual equipment and simulation system exchange information through sensors and actuators. The sensor provides the actual equipment information to the parallel simulation system, and the actuator lets the parallel simulation system to perform control and other operations on the actual equipment. The actual equipment information provided by the sensors can be divided into two categories, namely, observable state information *S*_*t*_ and behavior information *B*_*t*_ at time *t*. Equipment can be controlled by the control information *C*_*t*_ sent by the parallel simulation system. According to the implementation of control information, it can be divided into automatic control information AC_*t*_ and manual control information MC_*t*_.

An important concept in equipment parallel simulation is model evolution driven by the real-time data, including two aspects of model adaptive selection and model parameter evolution. This is considered to be a typical feature that distinguishes it from previous simulation techniques. In the past simulation technologies, the simulation model focused on one-time construction. After the simulation operation, the simulation model and model parameters no longer change, i.e., there is no model evolution process. Although the word “parallel” in parallel simulation translates as “parallel” in English, it is essentially different from “parallel” in parallel simulation proposed for many years. The former refers to expand the actual complex problem into the virtual space, and the actual complex problem is handled by the interaction between virtual space and actual space. It has the same connotation as “parallelism” in parallel system theory [28]. The latter refers to dividing the actual complex problem into several subproblems handled simultaneously [29]. Theoretical origins of parallel simulation are closely related to parallel system theory [28, 30], dynamic data drive application system (DDDAS) [31, 32], symbiotic simulation [33, 34], and online simulation [35]. The literature [29] has been reviewed in detail, but there are also significant differences. Parallel system theory emphasizes agent-based modeling. DDDAS introduces the idea of control theory into the simulation field, which stresses utilizing the simulation results to control the measurement process. Symbiotic simulation underlines using what-if analysis (WIA) to execute multiscenario simulation. Online simulation emphasizes the connecting relationship between the actual system and the simulation system, which is a contrary concept to offline simulation.

According to its technical principle and typical characteristics, equipment parallel simulation provides an effective way to solve the RUL prediction issue of hybrid degradation equipment. In the field of mechanical equipment, components such as bearings and gearboxes are widely used and they are all critical components. The failure of these components will cause the entire equipment to be shut down. Therefore, the performance degradation process of these components is often used to measure the RUL of mechanical equipment. The degradation process of these critical components is a typical hybrid degradation process. In this paper, with the background of RUL prediction issue of hybrid degradation equipment, the simulation model construction, model soft switch, model parameter evolution, and RUL prediction of hybrid degradation equipment RUL prediction oriented parallel simulation are investigated.

The structure of the paper is as follows. Based on the modeling analysis, the proposed simulation model is constructed in Section 2. The evolution method of the parallel simulation model is put forward in Section 3, which includes model probability based model soft switch and expectation maximization algorithm based model parameter evolution. Then, the RUL real-time prediction method of hybrid degradation equipment based on parallel simulation is presented in Section 4. The parallel simulation method given in this paper is validated by using a bearing degradation data, and comparative study is also conducted in Section 5. Finally, some conclusions and future perspectives are discussed in Section 6.

## 2. Modeling of Parallel Simulation

### 2.1. Modeling Analysis

The simulation model and its evolution belong to the model theory category of equipment parallel simulation, and it is also the basic issue in the research of equipment parallel simulation. Due to the strong field correlation of the model coupled with evolution characteristic, it becomes a difficult research issue in equipment parallel simulation. Therefore, it is the primary basic problem to determine the modeling method and model form in hybrid degradation equipment RUL prediction oriented parallel simulation.

Literatures [27, 36] pointed out that building a state space model for equipment performance degradation is an reasonable modeling direction for equipment RUL prediction oriented parallel simulation. In the SSM modeling approach, the simulation output is a hidden degradation state. The equipment degradation SSM includes a degradation state equation and an observation equation. The former describes the relationship between degradation states at adjacent moments, and the latter describes the relationship between the observation and degradation state. In the equipment degradation SSM, the dynamic and time-varying characteristics for the degradation process are both taken into consideration, which is helpful for the simulated degradation state estimation and RUL prediction. Considering that statistics-based methods are easier to obtain the analytical expression of the RUL probability density function, this paper combines the stochastic process approach with SSM to develop the parallel simulation model. The stochastic process is suitable for describing the randomness and uncertainty of the degradation process. The Wiener process and Gamma process are the most commonly used stochastic processes, but the application conditions of the latter are too harsh. The Gamma process is only suitable for describing the degradation processes with strictly monotonic characteristic. Contrarily, the Wiener process is suitable for describing the nonmonotonic degradation process, which has a more relaxed application conditions. Therefore, it is an advisable choice to construct the Wiener state space model (WSSM) by combining the SSM modeling method and the Wiener process in the parallel simulation modeling [36]. In particular, in order to describe the hybrid degradation process with unknown discrete shocks, a hybrid Wiener state space model (HWSSM) should be established as the parallel simulation model, which includes the continuous degradation model and the degradation model with unknown discrete shocks.

### 2.2. Construction of HWSSM

In order to construct HWSSM, two equations need to be developed, i.e., hybrid degradation state equation and observation equation. The Wiener process and shock effect are used to construct the hybrid degradation state equation with two forms, including the continuous degradation state equation and the degradation state equation with unknown discrete shock. The continuous degradation state equation can be formulated as

(1) $$\begin{array}{c} x\left(t\right)=x\left(0\right)+\eta t+\sigma B\left(t\right), \end{array}$$

where {*x*(*t*), *t* ≥ 0} is the continuous degradation process driven by the standard Brownian motion {*B*(*t*), *t* ≥ 0} and *B*(*t*) ~ *N*(0, *t*); *x*(0) is an initial degradation state; *η* and *σ* are the drift coefficient and diffusion coefficient of the standard Brownian motion [37]. Then, equation (1) is transformed by Euler discretization and the degradation state equation at discrete time points *t*_*k*_(*k*=1,2,…) without considering the unknown discrete shocks is yielded as

(2) $$\begin{array}{c} x_{k}=x_{k-1}+\eta \tau_{k}+\sigma \sqrt{\tau_{k}}\varpi_{k}, \end{array}$$

where *τ*_*k*_=*t*_*k*_ − *t*_*k*−1_ is the sensor sampling interval and *x*_*k*_=*x*(*t*_*k*_) is the simulated degradation state at time *t*_*k*_. Time *t*_*k*_ is short for time, *k* and *ϖ*_*k*_ is the noise sequence which obeys the standard normal distribution.

The effect of unknown discrete shocks on equipment performance can be represented by a shock variable. According to the discrete characteristic of the shocks, the damage caused by the shocks is integrated into equation (2), and a degradation state equation with discrete shocks is obtained by

(3) $$\begin{array}{c} x_{k}=x_{k-1}+\eta \tau_{k}+\sigma \sqrt{\tau_{k}}\varpi_{k}+D, \end{array}$$

where *D* is the damage caused by the shocks. We assume that the arrival of the shocks obeys the Poisson process [38, 39], i.e.,

(4) $$\begin{array}{c} P\left(M\left(t_{k}+\Delta t\right)-M\left(t_{k}\right)=n\right)=\frac{{\left(\rho \Delta t\right)}^{n}}{n!}e^{-\rho \Delta t}, \end{array}$$

where *M*(*t*_*k*_) denotes the total number of shocks that appear from the initial time until the moment *k* and *ρ* denotes the shocks arrival rate. Specifically, considering that the sampling interval of the sensor is small, the probability of more than one shock occurring within one sampling interval is very small. In other words, the probability of more than one shock occurring within one sampling interval can be neglected, and *n* is zero or one. In addition, it is assumed that the Poisson process is independent of the Brownian motion.

Equipment degradation observation data *y*(*t*) are obtained by sensor measurement. The random relationship between the simulated degradation state and the observation data can be described by the observation equation, i.e.,

(5) $$\begin{array}{c} y\left(t\right)=x\left(t\right)+\pi \left(t\right), \end{array}$$

where *π*(*t*) ~ *N*(0, *ϕ* ^2^) and *ϕ* ^2^ denotes the variance of the measurement noise. It is also assumed that *π*(*t*) is independent of the Brownian motion. Then, equation (5) is transformed by Euler discretization, and the observation equation at discrete time points *t*_*k*_(*k*=1,2,…) is yielded as

(6) $$\begin{array}{c} y_{k}=x_{k}+\phi \zeta_{k}, \end{array}$$

where *y*_*k*_=*y*(*t*_*k*_) denotes the degraded observation data at time *k* and *ζ*_*k*_ is the noise sequence that obeys the standard normal distribution. Furthermore, *ζ*_*k*_ is independent of *ϖ*_*k*_.

According to equations (1)–(6), the HWSSM at the discrete time point can be formulated as

(7) $$\begin{array}{c} \left\{\begin{array}{c} x_{k}=\left\{\begin{array}{cc} x_{k-1}+\eta \tau_{k}+\sigma \sqrt{\tau_{k}}\varpi_{k}, & \text{if}\;\;M\left(t_{k}\right)-M\left(t_{k-1}\right)=0,\\ x_{k-1}+\eta \tau_{k}+\sigma \sqrt{\tau_{k}}\varpi_{k}+D, & \text{if}\;\;M\left(t_{k}\right)-M\left(t_{k-1}\right)=1, \end{array}\right.\\ y_{k}=x_{k}+\phi \zeta_{k}. \end{array}\right. \end{array}$$

## 3. HWSSM Evolution

HWSSM belongs to the state space model with hidden degradation state. The parallel simulation system realizes the evolution of HWSSM by the following two ways. The first is the model soft switch based on the interactive multiple model (IMM) filtering [40, 41]. Through the IMM filtering, the probabilities of different simulation models are calculated dynamically, and the data assimilation between the observation data and the simulation output is achieved. As a result, the simulation output is updated and the estimation of the simulated degradation state is obtained. Another way is the model parameter evolution based on parameter online estimation. The model parameters are updated by utilizing the latest observation data via the parameter evolution. The model soft switch and parameter evolution are not executed in isolation, but they are iterative to each other. Through the iteration of the two ways, the simulation output is continuously approaching the equipment's actual degradation state. Finally, a high-fidelity simulation model is provided for accurately predicting the equipment RUL.

### 3.1. IMM Filtering-Based Model Soft Switch

In the HWSSM, the unknown characteristic of the shocks make it impossible to know whether there is a shock arrival from the observed data, which leads to no way to inform the conditions of switching the simulation model. Consequently, it is necessary to calculate the probabilities of different simulation models. In the SSM modeling method, the IMM filtering provides an effective way for calculating the probability of different models, and the Kalman filter is utilized in IMM filtering. The simulation model soft switch includes four stages, i.e., model input interaction, Kalman filtering, model probability calculation, and model output interaction.

For convenience, the following definitions are given. **Y**_*k*_={*y*_1_, *y*_2_,…, *y*_*k*_} and **X**_*k*_={*x*_0_, *x*_1_, *x*_2_,…, *x*_*k*_} represent the observation data vector and simulated degradation state vector until time *k*, respectively. *m* represents the number of simulation models. *μ*_*k*_^*u*^ represents the probability of simulation model *u* at time *k*. *p*_*vu*_ represents the transition probability from the model *v* to the model *u*. **P**=[*p*_*vu*_]_*m*×*m*_ represents the Markov probability transition matrix. *C*_*k*_^*u*^ denotes that the valid model is model *u* in time interval (*k* − 1, *k*]. ${\hat{x}}_{k\left|k\right.}^{u}$ denotes the estimated mean of the simulated degradation state at time *k* conditioned on *C*_*k*_^*u*^ and the previous *k* measured values. *P*_*k*|*k*_^*u*^ denotes the estimated covariance of the simulated degradation state at time *k* conditioned on *C*_*k*_^*u*^ and the previous *k* measured values. ${\hat{x}}_{k\left|k\right.}^{0u}$ denotes the hybrid estimated mean of the simulated degradation state at time *k* conditioned on *C*_*k*+1_^*u*^ and the previous *k* measured values. *P*_*k*|*k*_^0*u*^ denotes the hybrid estimated covariance of the simulated degradation state at time *k* conditioned on *C*_*k*+1_^*u*^ and the previous *k* measured values. *N*(*x*; 0, *κ*) denotes the Gaussian distribution with value *x*, mean 0, and variance *κ*.

#### 3.1.1. Input Interaction (Model *u*)

At this stage, the hybrid state estimation ${\hat{x}}_{k-1\left|k\right.-1}^{0u}$ and the covariance estimation *P*_*k*−1|*k*−1_^0*u*^ are obtained by the state estimation ${\hat{x}}_{k-1\left|k\right.-1}^{v}$ and model probability *μ*_*k*−1_^*v*^ of model *v* at time *k* − 1. ${\hat{x}}_{k-1\left|k\right.-1}^{0u}$ and *P*_*k*−1|*k*−1_^0*u*^ are both used as the initial state of the Kalman filtering at the time *k*. The specific steps are as follows.

The prior probability ${\bar{G}}_{u}$ of the simulation model *u* is defined and calculated by

(8) $$\begin{array}{c} {\bar{G}}_{u}\overset{\Delta }{=}p\left(\left.C_{k}^{u}\right|Y_{k-1}\right)=\overset{m}{\underset{v=1}{\sum }}p\left(\left.C_{k}^{u}\right|C_{k-1}^{v},Y_{k-1}\right)p\left(\left.C_{k-1}^{v}\right|Y_{k-1}\right)=\overset{m}{\underset{v=1}{\sum }}p_{vu}\mu_{k-1}^{v}, \end{array}$$

where $p_{vu}\overset{\Delta }{=}P\left(\left.C_{k}^{u}\right|C_{k-1}^{v},Y_{k-1}\right)$.

The hybrid probability *μ*_*k*−1|*k*−1_^*v*|*u*^ transferred from the model *v* to the model *u* is defined and calculated by

(9) $$\begin{array}{c} \mu_{k-1\left|k\right.-1}^{v\left|u\right.}\overset{\Delta }{=}p\left(\left.C_{k-1}^{v}\right|C_{k}^{u},Y_{k-1}\right)=\frac{p\left(\left.C_{k}^{u}\right|C_{k-1}^{v},Y_{k-1}\right)p\left(\left.C_{k-1}^{v}\right|Y_{k-1}\right)}{p\left(\left.C_{k}^{u}\right|Y_{k-1}\right)}=\frac{p_{vu}\mu_{k-1}^{v}}{{\bar{G}}_{u}}. \end{array}$$

The hybrid state estimation ${\hat{x}}_{k-1\left|k\right.-1}^{0u}$ of simulation model *u* is determined by

(10) $$\begin{array}{c} {\hat{x}}_{k-1\left|k-\right.1}^{0u}=\overset{m}{\underset{v=1}{\sum }}{\hat{x}}_{k-1\left|k\right.-1}^{v}\mu_{k-1\left|k\right.-1}^{v\left|u\right.}. \end{array}$$

The hybrid covariance estimation *P*_*k*−1|*k*−1_^0*u*^ of simulation model *u* is determined by

(11) $$\begin{array}{c} P_{k-1\left|k\right.-1}^{0u}=\overset{m}{\underset{v=1}{\sum }}\mu_{k-1\left|k\right.-1}^{v\left|u\right.}\left[P_{k-1\left|k\right.-1}^{v}+\left({\hat{x}}_{k-1\left|k\right.-1}^{v}-{\hat{x}}_{k-1\left|k\right.-1}^{0u}\right)\cdot {\left({\hat{x}}_{k-1\left|k\right.-1}^{v}-{\hat{x}}_{k-1\left|k\right.-1}^{0u}\right)}^{\text{T}}\right]. \end{array}$$

#### 3.1.2. Kalman Filtering (Model *u*)

Kalman filtering can be divided into two steps, i.e., the prediction step and the updating step. ${\hat{x}}_{k\left|k\right.-1}^{u}$ and *P*_*k*|*k*−1_^*u*^ express the simulated degradation state estimation and covariance estimation, respectively, conditioned on *C*_*k*_^*u*^ and the previous *k* − 1 measured values. Then, the prediction step refers to achieve the predicted results ${\hat{x}}_{k\left|k\right.-1}^{u}$ and *P*_*k*|*k*−1_^*u*^ according to the degradation equations and the results of input interaction. The prediction step can be expressed as

(12) $$\begin{array}{c} {\hat{x}}_{k\left|k\right.-1}^{1}={\hat{x}}_{k-1\left|k\right.-1}^{01}+\eta \tau_{k}, \end{array}$$

(13) $$\begin{array}{c} {\hat{x}}_{k\left|k\right.-1}^{2}={\hat{x}}_{k-1\left|k\right.-1}^{02}+\eta \tau_{k}+D, \end{array}$$

(14) $$\begin{array}{c} P_{k\left|k\right.-1}^{u}=P_{k-1\left|k\right.-1}^{0u}+\sigma^{2}\tau_{k}. \end{array}$$

The updating step refers to obtain the posterior state estimation ${\hat{x}}_{k\left|k\right.}^{u}$ and covariance estimation *P*_*k*|*k*_^*u*^ of model *u* based on the prognostic results in the prediction step and the observed data *y*_*k*_. The updating step can be expressed as

(15) $$\begin{array}{c} \left\{\begin{array}{c} {\tilde{y}}_{k}^{u}=y_{k}-{\hat{x}}_{k\left|k\right.-1}^{u},\\ S_{k}^{u}=P_{k\left|k\right.-1}^{u}+\phi^{2},\\ K_{k}^{u}=P_{k\left|k\right.-1}^{u}{\left(S_{k}^{u}\right)}^{-1},\\ {\hat{x}}_{k\left|k\right.}^{u}={\hat{x}}_{k\left|k\right.-1}^{u}+K_{k}^{u}{\tilde{y}}_{k}^{u},\\ P_{k\left|k\right.}^{u}=\left(I-K_{k}^{u}\right)P_{k\left|k\right.-1}^{u}, \end{array}\right. \end{array}$$

where ${\tilde{y}}_{k}^{u}$ denotes the new information, *S*_*k*_^*u*^ is the variance of ${\tilde{y}}_{k}^{u}$, *K*_*k*_^*u*^ is the Kalman gain, and *I* represents one-dimensional unit matrix.

#### 3.1.3. Model Probability Calculation

At this stage, the likelihood function Λ_*k*_^*u*^ of the model *u* is used to calculate the model probability and is defined as

(16) $$\begin{array}{c} \Lambda_{k}^{u}\overset{\Delta }{=}p\left(\left.y_{k}\right|C_{k}^{u},Y_{k-1}\right)\approx N\left[y_{k};{\hat{x}}_{k\left|k\right.-1}^{u},P_{k\left|k\right.-1}^{u}+\phi^{2}\right]. \end{array}$$

Furthermore, according to equation (16), Λ_*k*_^*u*^ can be given by

(17) $$\begin{array}{c} \Lambda_{k}^{u}=\frac{1}{{\left(2\pi \right)}^{1/2}{\left|S_{k}^{u}\right|}^{1/2}}\exp\left[-\frac{1}{2}{\left({\tilde{y}}_{k}^{u}\right)}^{\text{T}}{\left(S_{k}^{u}\right)}^{-1}{\tilde{y}}_{k}^{u}\right]. \end{array}$$

Then, the probability of the simulation model *u* is

(18) $$\begin{array}{c} \mu_{k}^{u}\overset{\Delta }{=}p\left(\left.C_{k}^{u}\right|Y_{k}\right)=\frac{\Lambda_{k}^{u}{\bar{G}}_{u}}{G_{u}}, \end{array}$$

where *G*_*u*_ is a normalization constant that satisfies $G_{u}=\sum_{u=1}^{m}\Lambda_{k}^{u}{\bar{G}}_{u}$.

#### 3.1.4. Output Interaction

According to the model probability *μ*_*k*_^*u*^ and the estimation results of each model in the Kalman filtering stage, the degradation state estimation ${\hat{x}}_{k\left|k\right.}$ and covariance estimation *P*_*k*|*k*_ are achieved through the weighted sum and can be given by

(19) $$\begin{array}{c} {\hat{x}}_{k\left|k\right.}=\overset{m}{\underset{u=1}{\sum }}{\hat{x}}_{k\left|k\right.}^{u}\mu_{k}^{u}, \end{array}$$

(20) $$\begin{array}{c} P_{k\left|k\right.}=\overset{m}{\underset{u=1}{\sum }}\mu_{k}^{u}\left[P_{k\left|k\right.}^{u}+\left({\hat{x}}_{k\left|k\right.}^{u}-{\hat{x}}_{k\left|k\right.}\right){\left({\hat{x}}_{k\left|k\right.}^{u}-{\hat{x}}_{k\left|k\right.}\right)}^{\text{T}}\right]. \end{array}$$

In addition, in the initial time of degraded state estimation, there exists *x*_0|0_^*u*^=*x*_0|0_^*v*^ and *p*(*C*_0_^*u*^)=*p*(*C*_0_^*v*^) conditioned on ∀*u*, *v* ∈ *m*, *u* ≠ *v*. Once a new measurement value *y*_*k*+1_ is obtained, the model probability can be updated to realize model soft switch according to equations (8)–(20).

### 3.2. Model Parameter Evolution

The model parameters ***θ*** of HWSSM include *μ*_0_, Σ_0_, *η*, *σ*, *D*, and *ϕ*, where *μ*_0_ and Σ_0_ denote the mean and covariance of the initial simulated degradation state, respectively. Since HWSSM is a state space model with hidden state, it is considered to use the expectation maximization (EM) algorithm to achieve model parameter evolution. EM algorithm is suitable for estimating model parameters of SSM with hidden state [42, 43]. Based on the model soft switch, this paper uses EM algorithm to realize the evolution of model parameters.

According to the EM algorithm, the estimations of the model parameters ***θ*** at time *k* and the *j*th iteration of EM algorithm can be obtained by

(21) $$\begin{array}{c} {\hat{\theta }}_{k}^{\left(j\right)}=\arg\underset{\theta }{\max}E_{X_{k},{\tilde{R}}_{1:k}\left|Y_{k}\right.,{\hat{\theta }}_{k}^{\left(j-1\right)}}\left(\left.L\left(\left.X_{k},Y_{k},{\tilde{R}}_{1:k}\right|\theta \right)\right|Y_{k},{\hat{\theta }}_{k}^{\left(j-1\right)}\right), \end{array}$$

where *E*(·) denotes the mathematical expectation operator, $L\left(\left.X_{k},Y_{k},{\tilde{R}}_{1:k}\right|\theta \right)$ is the joint log-likelihood function, and ${\tilde{R}}_{1:k}$ represents the model indicating matrix. ${\tilde{R}}_{k}$ represents the model indicating vector at time *k* and satisfies ${\tilde{R}}_{k}=\left\{{\tilde{R}}_{k}^{1},{\tilde{R}}_{k}^{2}\right\}$. Its element ${\tilde{R}}_{k}^{v}$ is defined as

(22) $$\begin{array}{c} {\tilde{R}}_{k}^{v}=\left\{\begin{array}{cc} 1, & \text{conditioned on }C_{k}^{v},\\ 0, & \text{others}. \end{array}\right. \end{array}$$

EM algorithm consists of two steps, i.e., E-step and M-step. E-step refers to calculate the mathematical expectation of the joint log-likelihood function, and M-step refers to maximize the mathematical expectation of the joint log-likelihood function. The model parameters can be estimated online via the iteration of E-step and M-step.

#### 3.2.1. E-Step

Based on the Markov property and the multiplicative equation of conditional probability, the joint log-likelihood function of the simulated state vector **X**_*k*_, the observed data vector **Y**_*k*_, and the model indicating matrix ${\tilde{R}}_{1:k}$ at time *k* is given as

(23) $$\begin{array}{c} L\left(\left.X_{k},Y_{k},{\tilde{R}}_{1:k}\right|\theta \right)=\ln\;\;p\left(\left.X_{k},Y_{k},{\tilde{R}}_{1:k}\right|\theta \right)=\ln\left(p\left(\left.Y_{k}\right|X_{k},\theta \right)p\left(\left.X_{k},{\tilde{R}}_{1:k}\right|\theta \right)\right)\\ =\underset{①}{\underset{︸}{\ln\;\;p\left(\left.Y_{k}\right|X_{k},\theta \right)}}+\underset{②}{\underset{︸}{\ln\;\;p\left(\left.X_{k},{\tilde{R}}_{1:k}\right|\theta \right)}}. \end{array}$$

According to HWSSM, there exists

(24) $$\begin{array}{c} p\left(\left.y_{i}\right|x_{i},\theta \right)=N\left(y_{i};x_{i},\phi^{2}\right);p\left(\left.x_{i}\right|x_{i-1},{\tilde{R}}_{i}^{v},{\tilde{R}}_{1:i-1},\theta \right)=N\left(x_{i};x_{i-1}+\chi_{i}^{v},\sigma^{2}\tau_{i}\right);p\left(\left.x_{0}\right|\theta \right)=N\left(x_{0};\mu_{0},\Sigma_{0}\right), \end{array}$$

where *χ*_*i*_^1^=*ητ*_*i*_ and *χ*_*i*_^2^=*ητ*_*i*_+*D*. Then, the derivation of equation (23) is as follows:

(25) $$\begin{array}{c} ①=\ln\overset{k}{\underset{i=1}{\prod }}p\left(\left.y_{i}\right|x_{i},\theta \right)=\overset{k}{\underset{i=1}{\sum }}\ln\;\;p\left(\left.y_{i}\right|x_{i},\theta \right)=\overset{k}{\underset{i=1}{\sum }}\ln\left(\frac{1}{\sqrt{2\pi \phi^{2}}}\exp\left(-\frac{{\left(y_{i}-x_{i}\right)}^{2}}{2\phi^{2}}\right)\right)\\ =-\frac{k}{2}\ln\;\;2\;\;\pi -\frac{k}{2}\ln\;\;\phi^{2}-\frac{1}{2\phi^{2}}\overset{k}{\underset{i=1}{\sum }}{\left(y_{i}-x_{i}\right)}^{2},\\ ②=\ln\left\{\overset{k}{\underset{i=2}{\prod }}p\left(x_{i},\left.{\tilde{R}}_{i}\right|x_{i-1},{\tilde{R}}_{1:i-1},\theta \right)p\left(x_{1},\left.{\tilde{R}}_{1}\right|x_{0},\theta \right)p\left(\left.x_{0}\right|\theta \right)\right\}\\ =\ln\left\{p\left(\left.x_{0}\right|\theta \right)\overset{k}{\underset{i=1}{\prod }}\overset{2}{\underset{v=1}{\prod }}{\left[\mu_{i}^{v}N\left(x_{i};x_{i-1}+\chi_{i}^{v},\sigma^{2}\tau_{i}\right)\right]}^{{\tilde{R}}_{i}^{v}}\right\}\\ =\underset{③}{\underset{︸}{\ln\;\;p\left(\left.x_{0}\right|\theta \right)}}+\underset{④}{\underset{︸}{\ln\left\{\overset{k}{\underset{i=1}{\prod }}\overset{2}{\underset{v=1}{\prod }}{\left[\mu_{i}^{v}N\left(x_{i};x_{i-1}+\chi_{i}^{v},\sigma^{2}\tau_{i}\right)\right]}^{{\tilde{R}}_{i}^{v}}\right\}}},\\ ③=\ln\;\;p\left(\left.x_{0}\right|\theta \right)=-\frac{1}{2}\ln\;\;2\pi -\frac{1}{2}\ln\;\;\Sigma_{0}-\frac{{\left(x_{0}-\mu_{0}\right)}^{2}}{2\Sigma_{0}},\\ ④=\overset{k}{\underset{i=1}{\sum }}\overset{2}{\underset{v=1}{\sum }}\left\{\ln\;\;{\left[\mu_{i}^{v}N\left(x_{i};x_{i-1}+\chi_{i}^{v},\sigma^{2}\tau_{i}\right)\right]}^{{\tilde{R}}_{i}^{v}}\right\}\\ =\overset{k}{\underset{i=1}{\sum }}\overset{2}{\underset{v=1}{\sum }}\left\{{\tilde{R}}_{i}^{v}\ln\left[\mu_{i}^{v}N\left(x_{i};x_{i-1}+\chi_{i}^{v},\sigma^{2}\tau_{i}\right)\right]\right\}\\ =\overset{2}{\underset{v=1}{\sum }}\left\{\overset{k}{\underset{i=1}{\sum }}{\tilde{R}}_{i}^{v}\left\{\ln\;\;\mu_{i}^{v}+\ln\;\;N\left(x_{i};x_{i-1}+\chi_{i}^{v},\sigma^{2}\tau_{i}\right)\right\}\right\}\\ =\overset{2}{\underset{v=1}{\sum }}\left\{\overset{k}{\underset{i=1}{\sum }}{\tilde{R}}_{i}^{v}\left\{\ln\;\;\mu_{i}^{v}+\ln\frac{1}{\sqrt{2\pi \tau_{i}}}-\ln\;\;\sigma -\frac{1}{2\sigma^{2}\tau_{i}}{\left[x_{i}-\left(x_{i-1}+\chi_{i}^{v}\right)\right]}^{2}\right\}\right\}. \end{array}$$

Based on the above derivation, the final expression of the joint log-likelihood function is formulated as

(26) $$\begin{array}{c} L\left(X_{k},Y_{k},\left.{\tilde{R}}_{1:k}\right|\theta \right)=-\frac{k}{2}\ln\;\;2\pi -\frac{k}{2}\ln\;\;\phi^{2}-\frac{1}{2\phi^{2}}\overset{k}{\underset{i=1}{\sum }}{\left(y_{i}-x_{i}\right)}^{2}+\overset{2}{\underset{v=1}{\sum }}\left\{\overset{k}{\underset{i=1}{\sum }}{\tilde{R}}_{i}^{v}\left\{\ln\;\;\mu_{i}^{v}+\ln\frac{1}{\sqrt{2\pi \tau_{i}}}-\ln\;\;\sigma -\frac{1}{2\sigma^{2}\tau_{i}}{\left[x_{i}-\left(x_{i-1}+\chi_{i}^{v}\right)\right]}^{2}\right\}\right\}-\frac{1}{2}\ln\;\;2\pi -\frac{1}{2}\ln\;\;\Sigma_{0}-\frac{{\left(x_{0}-\mu_{0}\right)}^{2}}{2\Sigma_{0}}. \end{array}$$

Next, *Q*(***θ***, ***θ***_*k*_^(*j*)^) is denoted as the mathematical expectation of the joint log-likelihood function at time *k* and the *j*th iteration of EM algorithm, i.e.,

(27) $$\begin{array}{c} Q\left(\theta ,\theta_{k}^{\left(j\right)}\right)=E_{X_{k},{\tilde{R}}_{1:k}\left|Y_{k}\right.,{\hat{\theta }}_{k}^{\left(j-1\right)}}\left(\left.L\left(X_{k},Y_{k},\left.{\tilde{R}}_{1:k}\right|\theta \right)\right|Y_{k},{\hat{\theta }}_{k}^{\left(j-1\right)}\right). \end{array}$$

Through neglecting the irrelevant items that are independent of the parameter ***θ***, *Q*(***θ***, ***θ***_*k*_^(*j*)^) can be expressed as

(28) $$\begin{array}{c} Q\left(\theta ,\theta_{k}^{\left(j\right)}\right)\propto -\overset{k}{\underset{i=1}{\sum }}\left(\frac{1}{2}\ln\;\;\phi^{2}+\frac{1}{2\phi^{2}}\underset{⑤}{\underset{︸}{E_{X_{k},\left.{\tilde{R}}_{1:k}\right|\cdot }\left(\left.{\left(y_{i}-x_{i}\right)}^{2}\right|\cdot \right)}}\right)+\overset{2}{\underset{v=1}{\sum }}\left\{\overset{k}{\underset{i=1}{\sum }}\underset{⑥}{\underset{︸}{E_{X_{k},\left.{\tilde{R}}_{1:k}\right|\cdot }\left(\left.{\tilde{R}}_{i}^{v}\right|\cdot \right)}}\left\{\ln\;\;\mu_{i}^{v}+\ln\frac{1}{\sqrt{2\pi \tau_{i}}}-\ln\;\;\sigma -\frac{\underset{⑦}{\underset{︸}{E_{X_{k},\left.{\tilde{R}}_{1:k}\right|\cdot }\left[\left.{\left(x_{i}-x_{i-1}-\chi_{i}^{v}\right)}^{2}\right|\cdot \right]}}}{2\sigma^{2}\tau_{i}}\right\}\right\}-\frac{1}{2}\ln\;\;\Sigma_{0}-\frac{1}{2\Sigma_{0}}\underset{⑧}{\underset{︸}{E_{X_{k},\left.{\tilde{R}}_{1:k}\right|\cdot }\left(\left.{\left(x_{0}-\mu_{0}\right)}^{2}\right|Y_{k},{\hat{\theta }}_{k}^{\left(j-1\right)}\right)}}, \end{array}$$

where · represents $Y_{k},{\hat{\theta }}_{k}^{\left(j-1\right)}$. According to the property of mathematical expectation, the following intermediate variables are defined as

(29) $$\begin{array}{c} {\hat{x}}_{i\left|k\right.}=E_{X_{k},{\tilde{R}}_{1:k}\left|\cdot \right.}\left(\left.x_{i}\right|\cdot \right),\\ P_{i\left|k\right.}={\mathrm{var}}_{X_{k},{\tilde{R}}_{1:k}\left|\cdot \right.}\left(\left.x_{i}^{2}\right|\cdot \right)=E_{X_{k},{\tilde{R}}_{1:k}\left|\cdot \right.}\left(\left.x_{i}^{2}\right|\cdot \right)-{\left(E_{X_{k},{\tilde{R}}_{1:k}\left|\cdot \right.}\left(\left.x_{i}\right|\cdot \right)\right)}^{2}=E_{X_{k},{\tilde{R}}_{1:k}\left|\cdot \right.}\left(\left.x_{i}^{2}\right|\cdot \right)-{\hat{x}}_{i\left|k\right.}^{2},\\ P_{i,i-1\left|k\right.}={\mathrm{cov}}_{X_{k},{\tilde{R}}_{1:k}\left|\cdot \right.}\left(\left.x_{i}x_{i-1}\right|\cdot \right)=E_{X_{k},{\tilde{R}}_{1:k}\left|\cdot \right.}\left(\left.x_{i}x_{i-1}\right|\cdot \right)-E_{X_{k},{\tilde{R}}_{1:k}\left|\cdot \right.}\left(\left.x_{i}\right|\cdot \right)E_{X_{k},{\tilde{R}}_{1:k}\left|\cdot \right.}\left(\left.x_{i-1}\right|\cdot \right)\\ =E_{X_{k},{\tilde{R}}_{1:k}\left|\cdot \right.}\left(\left.x_{i}x_{i-1}\right|\cdot \right)-{\hat{x}}_{i\left|k\right.}{\hat{x}}_{i-1\left|k\right.}. \end{array}$$

Based on the definition of the above variables, the derivation of the four conditional mathematical expectations in equation (28) is as follows:

(30) $$\begin{array}{c} ⑤=E_{X_{k},{\tilde{R}}_{1:k}\left|\cdot \right.}\left(\left.y_{i}^{2}+x_{i}^{2}-2y_{i}x_{i}\right|\cdot \right)=y_{i}^{2}+E_{X_{k},{\tilde{R}}_{1:k}\left|\cdot \right.}\left(\left.x_{i}^{2}\right|\cdot \right)-2y_{i}E_{X_{k},{\tilde{R}}_{1:k}\left|\cdot \right.}\left(\left.x_{i}\right|\cdot \right)=y_{i}^{2}+P_{i\left|k\right.}+{\hat{x}}_{i\left|k\right.}^{2}-2y_{i}{\hat{x}}_{i\left|k\right.},\\ ⑥=E_{X_{k},{\tilde{R}}_{1:k}\left|\cdot \right.}\left(\left.{\tilde{R}}_{i}^{v}\right|\cdot \right)=p\left(\left.{\tilde{R}}_{i}^{v}=1\right|\cdot \right)=\omega_{i}^{v},\\ ⑦=E_{X_{k},{\tilde{R}}_{1:k}\left|\cdot \right.}\left[\left.{\left(x_{i}-x_{i-1}-\chi_{i}^{v}\right)}^{2}\right|\cdot \right]=E_{X_{k},{\tilde{R}}_{1:k}\left|\cdot \right.}\left(\left.\left({\left(\chi_{i}^{v}\right)}^{2}+{\left(x_{i}-x_{i-1}\right)}^{2}-2\chi_{i}^{v}\left(x_{i}-x_{i-1}\right)\right)\right|\cdot \right)\\ ={\left(\chi_{i}^{v}\right)}^{2}+{\hat{x}}_{i\left|k\right.}^{2}+P_{i\left|k\right.}+{\hat{x}}_{i-1\left|k\right.}^{2}+P_{i-1\left|k\right.}-2\left({\hat{x}}_{i\left|k\right.}{\hat{x}}_{i-1\left|k\right.}+P_{i,i-1\left|k\right.}\right)-2\chi_{i}^{v}\left({\hat{x}}_{i\left|k\right.}-{\hat{x}}_{i-1\left|k\right.}\right),\\ ⑧=E_{X_{k},{\tilde{R}}_{1:k}\left|\cdot \right.}\left(\left.{\left(x_{0}-\mu_{0}\right)}^{2}\right|\cdot \right)=E_{X_{k},{\tilde{R}}_{1:k}\left|\cdot \right.}\left(\left.x_{0}^{2}+\mu_{0}^{2}-2\mu_{0}x_{0}\right|\cdot \right)={\hat{x}}_{0\left|k\right.}^{2}+P_{0\left|k\right.}+\mu_{0}^{2}-2\mu_{0}{\hat{x}}_{0\left|k\right.}. \end{array}$$

After deriving the above four parts of the conditional mathematical expectation, *Q*(***θ***, ***θ***_*k*_^(*j*)^) can be given by

(31) $$\begin{array}{c} Q\left(\theta ,\theta_{k}^{\left(j\right)}\right)\propto -\overset{k}{\underset{i=1}{\sum }}\left(\frac{1}{2}\ln\;\;\phi^{2}+\frac{1}{2\phi^{2}}\left(y_{i}^{2}+P_{i\left|k\right.}+{\hat{x}}_{i\left|k\right.}^{2}-2y_{i}{\hat{x}}_{i\left|k\right.}\right)\right)+\overset{2}{\underset{v=1}{\sum }}\left\{\overset{k}{\underset{i=1}{\sum }}\omega_{i}^{v}\left\{\ln\;\;\mu_{i}^{v}+\ln\frac{1}{\sqrt{2\pi \tau_{i}}}-\ln\;\;\sigma -\frac{\left({\left(\chi_{i}^{v}\right)}^{2}+\Gamma_{i}-2\chi_{i}^{v}\left({\hat{x}}_{i\left|k\right.}-{\hat{x}}_{i-1\left|k\right.}\right)\right)}{2\sigma^{2}\tau_{i}}\right\}\right\}-\frac{1}{2}\ln\;\;\Sigma_{0}-\frac{1}{2\Sigma_{0}}\left({\hat{x}}_{0\left|k\right.}^{2}+P_{0\left|k\right.}+\mu_{0}^{2}-2\mu_{0}{\hat{x}}_{0\left|k\right.}\right), \end{array}$$

where $\Gamma_{i}={\hat{x}}_{i\left|k\right.}^{2}+P_{i\left|k\right.}+{\hat{x}}_{i-1\left|k\right.}^{2}+P_{i-1\left|k\right.}-2\left({\hat{x}}_{i\left|k\right.}{\hat{x}}_{i-1\left|k\right.}+P_{i,i-1\left|k\right.}\right)$. According to equation (31), in order to calculate *Q*(***θ***, ***θ***_*k*_^(*j*)^), the values of the variables ${\hat{x}}_{i\left|k\right.}$, ${\hat{x}}_{i-1\left|k\right.}$, *P*_*i*|*k*_, *P*_*i*−1|*k*_, *P*_*i*,*i*−1|*k*_, and *ω*_*i*_^*v*^ should be acquired, which belong to the smoothed variables. This paper uses the IMM backward smoothing (IMMBS) algorithm to obtain the values of the above six smoothed variables [44, 45].

IMMBS algorithm consists of backward filtering and model state fusion. Backward filtering refers to the backward filtering from the latest measured value. The operation flow of backward filtering is similar to the forward IMM filtering mentioned in Section 3.1, but there are also obvious differences between them. The forward IMM filtering performs the input interaction before performing one-step prediction, while the backward filtering performs one-step prediction before performing the input interaction. Backward filtering can be divided into five steps, including backward one-step prediction, backward input interaction, backward filtering update, backward model probability calculation, and backward output fusion. In particular, the backward one-step prediction equation for backward filtering is given by

(32) $$\begin{array}{c} {\hat{x}}_{i\left|i\right.+1}^{B,1}={\hat{x}}_{i+1\left|i\right.+1}^{B,1}-\eta \tau_{i+1},\\ {\hat{x}}_{i\left|i\right.+1}^{B,2}={\hat{x}}_{i+1\left|i\right.+1}^{B,2}-\eta \tau_{i+1}-D,\\ P_{i\left|i\right.+1}^{B,u}=P_{i+1\left|i\right.+1}^{B,u}+\sigma^{2}\tau_{i+1}, \end{array}$$

where ${\hat{x}}_{i\left|i\right.+1}^{B,u}$ denotes the backward one-step prediction of the model *u* and *P*_*i*|*i*+1_^*B*,*u*^ represents the covariance of backward one-step prediction error for the model *u*. The other steps of backward filtering can be found in [44]. In the stage of model state fusion for the IMMBS algorithm, according to the full probability theorem, the smoothing estimation of the simulated state can be expressed as

(33) $$\begin{array}{c} p\left(\left.x_{i}\right|Y_{k}\right)=\overset{m}{\underset{u=1}{\sum }}\omega_{i}^{u}p\left(\left.x_{i}\right|C_{i}^{u},Y_{k}\right)=\overset{m}{\underset{u=1}{\sum }}\omega_{i}^{u}\left[\overset{m}{\underset{v=1}{\sum }}{\tilde{\omega }}_{i+1\left|k\right.}^{v\left|u\right.}p\left(\left.x_{i}\right|C_{i}^{u},C_{i+1}^{v},Y_{k}\right)\right]=\overset{m}{\underset{u=1}{\sum }}\omega_{i}^{u}\left[\overset{m}{\underset{v=1}{\sum }}{\tilde{\omega }}_{i+1\left|k\right.}^{v\left|u\right.}\Lambda_{i}^{uv}\right], \end{array}$$

where *ω*_*i*_^*u*^ represents the smoothed model probability, ${\tilde{\omega }}_{i+1\left|k\right.}^{v\left|u\right.}$ denotes the smoothed hybrid probability, and Λ_*i*_^*uv*^ denotes the likelihood function. The definition and calculation of the three variables are given by

(34) $$\begin{array}{c} {\tilde{\omega }}_{i+1\left|k\right.}^{v\left|u\right.}\overset{\Delta }{=}p\left(\left.C_{i+1}^{v}\right|C_{i}^{u},Y_{1}^{k}\right)=\frac{1}{{\tilde{G}}_{u}}p\left(\left.C_{i+1}^{v}\right|C_{i}^{u},Y_{i}\right)p\left(\left.Y_{i+1}^{k}\right|C_{i+1}^{v},C_{i}^{u},Y_{i}\right)=\frac{1}{{\tilde{G}}_{u}}p_{uv}p\left(\left.Y_{i+1}^{k}\right|C_{i+1}^{v},C_{i}^{u},Y_{i}\right),\\ \Lambda_{i}^{uv}\overset{\Delta }{=}p\left(\left.Y_{i+1}^{k}\right|C_{i+1}^{v},C_{i}^{u},Y_{i}\right)\approx p\left(\left.x_{i\left|i\right.+1}^{B,v}\right|C_{i+1}^{v},C_{i}^{u},x_{i\left|i\right.}^{u}\right)=N\left[{\hat{x}}_{i\left|i\right.+1}^{B,v}-{\hat{x}}_{i\left|i\right.}^{u};0,P_{i\left|i\right.+1}^{B,v}+P_{i\left|i\right.}^{u}\right],\\ \omega_{i}^{u}\overset{\Delta }{=}p\left(\left.C_{i}^{u}\right|Y_{1}^{k}\right)=\frac{{\tilde{G}}_{u}}{G}p\left(\left.C_{i}^{u}\right|Y_{i}\right)=\frac{{\tilde{G}}_{u}}{G}\mu_{i}^{u}, \end{array}$$

where ${\tilde{G}}_{u}$ and *G* are all the normalized constants. They can be acquired by

(35) $$\begin{array}{c} {\tilde{G}}_{u}=\overset{m}{\underset{v=1}{\sum }}p_{uv}\Lambda_{i}^{uv},\\ G=\overset{m}{\underset{u=1}{\sum }}{\tilde{G}}_{u}\mu_{i}^{u}. \end{array}$$

In equation (33), with respect to *p*(*x*_*i*_|*C*_*i*_^*u*^, *C*_*i*+1_^*v*^, **Y**_*k*_), the mixed smoothing state estimation ${\hat{x}}_{i\left|k\right.}^{uv}$, covariance estimation *P*_*i*|*k*_^*uv*^, and the mixed interaction covariance estimation *P*_*i*,*i*−1|*k*_^*uv*^ can be obtained by

(36) $$\begin{array}{c} {\hat{x}}_{i\left|k\right.}^{uv}=P_{i\left|k\right.}^{uv}\left[{\left(P_{i\left|i\right.}^{u}\right)}^{-1}{\hat{x}}_{i\left|i\right.}^{u}+{\left(P_{i\left|i\right.+1}^{B,v}\right)}^{-1}{\hat{x}}_{i\left|i\right.+1}^{B,v}\right],\\ P_{i\left|k\right.}^{uv}={\left[{\left(P_{i\left|i\right.}^{u}\right)}^{-1}+{\left(P_{i\left|i\right.+1}^{B,v}\right)}^{-1}\right]}^{-1},\\ P_{i,i-1\left|k\right.}^{uv}={\left\{{\left[{\left(P_{i\left|i\right.}^{u}\right)}^{-1}+{\left(P_{i\left|i\right.+1}^{B,v}\right)}^{-1}\right]}^{-1}+{\left[{\left(P_{i-1\left|i\right.-1}^{u}\right)}^{-1}+{\left(P_{i-1\left|i\right.}^{B,v}\right)}^{-1}\right]}^{-1}\right\}}^{-1}. \end{array}$$

In equation (33), with respect to *p*(*x*_*i*_|*C*_*i*_^*u*^, **Y**_*k*_), the mixed smoothing state estimation ${\hat{x}}_{i\left|k\right.}^{u}$, covariance estimation *P*_*i*|*k*_^*u*^, and the mixed interaction covariance estimation *P*_*i*,*i*−1|*k*_^*u*^ of model *u* can be obtained by

(37) $$\begin{array}{c} {\hat{x}}_{i\left|k\right.}^{u}=\overset{m}{\underset{u=1}{\sum }}{\tilde{\omega }}_{i+1\left|k\right.}^{v\left|u\right.}{\hat{x}}_{i\left|k\right.}^{uv},\\ P_{i\left|k\right.}^{u}=\overset{m}{\underset{u=1}{\sum }}{\tilde{\omega }}_{i+1\left|k\right.}^{v\left|u\right.}\left[P_{i\left|k\right.}^{uv}+\left({\hat{x}}_{i\left|k\right.}^{uv}-{\hat{x}}_{i\left|k\right.}^{u}\right){\left({\hat{x}}_{i\left|k\right.}^{uv}-{\hat{x}}_{i\left|k\right.}^{u}\right)}^{\text{T}}\right],\\ P_{i,i-1\left|k\right.}^{u}=\overset{m}{\underset{u=1}{\sum }}{\tilde{\omega }}_{i+1\left|k\right.}^{v\left|u\right.}\left[P_{i,i-1\left|k\right.}^{uv}+\left({\hat{x}}_{i\left|k\right.}^{uv}-{\hat{x}}_{i\left|k\right.}^{u}\right){\left({\hat{x}}_{i-1\left|k\right.}^{uv}-{\hat{x}}_{i-1\left|k\right.}^{u}\right)}^{\text{T}}\right]. \end{array}$$

In particular, if *i*=*k*, there exists

(38) $$\begin{array}{c} P_{k,k-1\left|k\right.}^{u}=\left(I-K_{k}^{u}\right)P_{k-1\left|k\right.-1}^{u}. \end{array}$$

Finally, after the smoothed state fusion of each model, the smooth estimation ${\hat{x}}_{i\left|k\right.}$, covariance estimation *P*_*i*|*k*_, and interactive covariance estimation *P*_*i*,*i*−1|*k*_ of the simulated degradation state can be acquired by

(39) $$\begin{array}{c} {\hat{x}}_{i\left|k\right.}=\overset{m}{\underset{u=1}{\sum }}\omega_{i}^{u}{\hat{x}}_{i\left|k\right.}^{u},\\ P_{i\left|k\right.}=\overset{m}{\underset{u=1}{\sum }}\omega_{i}^{u}\left[P_{i\left|k\right.}^{u}+\left({\hat{x}}_{i\left|k\right.}^{u}-{\hat{x}}_{i\left|k\right.}\right){\left({\hat{x}}_{i\left|k\right.}^{u}-{\hat{x}}_{i\left|k\right.}\right)}^{\text{T}}\right],\\ P_{i,i-1\left|k\right.}=\overset{m}{\underset{u=1}{\sum }}\omega_{i}^{u}\left[P_{i,i-1\left|k\right.}^{u}+\left({\hat{x}}_{i\left|k\right.}^{u}-{\hat{x}}_{i\left|k\right.}\right){\left({\hat{x}}_{i-1\left|k\right.}^{u}-{\hat{x}}_{i-1\left|k\right.}\right)}^{\text{T}}\right]. \end{array}$$

In particular, the initial smoothed estimations are given by

(40) $$\begin{array}{c} {\hat{x}}_{0\left|k\right.}=\overset{m}{\underset{u=1}{\sum }}\omega_{1}^{u}{\hat{x}}_{0\left|1\right.}^{B,u},\\ P_{0\left|k\right.}=\overset{m}{\underset{u=1}{\sum }}\omega_{1}^{u}\left[P_{0\left|1\right.}^{B,u}+\left({\hat{x}}_{0\left|1\right.}^{B,u}-{\hat{x}}_{0\left|k\right.}\right){\left({\hat{x}}_{0\left|1\right.}^{B,u}-{\hat{x}}_{0\left|k\right.}\right)}^{\text{T}}\right]. \end{array}$$

#### 3.2.2. M-Step

The estimation of the model parameters ***θ*** at the *j*th step of the EM algorithm can be obtained by differentiating equation (31), i.e.,

(41) $$\begin{array}{c} \frac{\partial Q\left(\theta ,\theta_{k}^{\left(j\right)}\right)}{\partial \theta }=0. \end{array}$$

The updated parameters are given by

(42) $$\begin{array}{c} \left\{\begin{array}{c} {\hat{\mu }}_{0}={\hat{x}}_{0\left|k\right.},\\ {\hat{\Sigma }}_{0}=P_{0\left|k\right.},\\ \hat{D}=\frac{\sum_{i=1}^{k}\omega_{i}^{2}\left({\hat{x}}_{i\left|k\right.}-{\hat{x}}_{i-1\left|k\right.}-\eta \tau_{i}\right)/\tau_{i}}{\sum_{i=1}^{k}\omega_{i}^{2}/\tau_{i}},\\ \hat{\eta }==\frac{\sum_{i=1}^{k}\left({\hat{x}}_{i\left|k\right.}-{\hat{x}}_{i-1\left|k\right.}-D\omega_{i}^{2}\right)}{\sum_{i=1}^{k}\tau_{i}},\\ {\hat{\sigma }}^{2}=\frac{1}{k}\overset{k}{\underset{i=1}{\sum }}\frac{\left({\left(\eta \tau_{i}\right)}^{2}+\omega_{i}^{2}\left(2\eta \tau_{i}D+D^{2}\right)+\Gamma_{i}-2\left(\eta \tau_{i}+\omega_{i}^{2}D\right)\left({\hat{x}}_{i\left|k\right.}-{\hat{x}}_{i-1\left|k\right.}\right)\right)}{\tau_{i}},\\ {\hat{\phi }}^{2}=\frac{1}{k}\overset{k}{\underset{i=1}{\sum }}\left(y_{i}^{2}+P_{i\left|k\right.}+{\hat{x}}_{i\left|k\right.}^{2}-2y_{i}{\hat{x}}_{i\left|k\right.}\right), \end{array}\right. \end{array}$$

where *ω*_*i*_^2^ denotes the smoothed probability of the model formulated by equation (3). Note that the updated equation of parameter *D* contains parameter *η*, the updated equation of parameter *η* contains parameter *D*, and the updated equation of parameter *σ* contains parameters *η* and *D*, which makes it impossible to directly use equation (42) to obtain the estimated values of parameters *D*, *η*, and *σ*. For this reason, the simplex method of multidimensional search in [36] is utilized to achieve the estimated values of three parameters, which is integrated as the “fminsearch” function in MATLAB [46]. The function is an effective method to search for the minimum value of the multidimensional function. The specific solving process is as follows. Equation (42) is taken into equation (31) to get an expression that only contains the parameters *D* and *η*. Then, the “fminsearch” function is used to perform a two-dimensional search starting from the initial value of the parameters *D* and *η*. When the expression −*Q*(***θ***, ***θ***_*k*_^(*j*)^) gets the minimum value, the expression *Q*(***θ***, ***θ***_*k*_^(*j*)^) obtains the maximum value. In this case, the corresponding parameter values are the estimated values of *D* and *η*. Finally, the estimated values of *D* and *η* are brought into the updated equation of the parameter *σ* in equation (42), and the estimated values of *σ* is achieved.

The abovementioned solving method can obtain the estimated values of model parameters at the *j*th iteration of EM algorithm. In other words, the one iteration from ${\hat{\theta }}_{k}^{\left(j-1\right)}$ to ${\hat{\theta }}_{k}^{\left(j\right)}$ is completed. Then, the estimated values are taken into IMM filtering-based model soft switch to update the model probabilities and the simulated degradation state and EM algorithm is performed again until a criterion of convergence is satisfied.

## 4. Parallel Simulation-Based Equipment RUL Real-Time Prediction

According to the widely used concept of first hitting time (FHT), the definition of the equipment RUL is given. Assuming that the equipment failure threshold is *w*, the equipment RUL *T* is defined as the time when the degradation process first passes the failure threshold [47], i.e.,

(43) $$\begin{array}{c} T\left(w\right)=\inf\left\{t:x\left(t\right)\geq \left.w\right|x\left(0\right)<w\right\}. \end{array}$$

In order to obtain the analytical expression of the PDF of RUL, the derivation of the RUL calculation at time *k* is performed below. Considering that there may be two degraded state equations at a particular moment in the HWSSM, the RUL probability density distribution cannot be obtained directly, so the degradation process *x*(*t*) needs to be transformed. At the current monitoring time *k*, the simulated degradation state is *x*_*k*_. According to the Markov characteristic of the Brownian motion, the Wiener process can be rewritten as

(44) $$\begin{array}{c} x\left(t\right)=x_{k}+\eta \left(t-t_{k}\right)+\sigma \left(B\left(t\right)-B\left(t_{k}\right)\right)=x_{k}+\eta \left(t-t_{k}\right)+\sigma B\left(t-t_{k}\right). \end{array}$$

Then the two state equations of HWSSM are merged into one equation. In other words, the damages caused by *n* Poisson shocks are integrated into the Wiener process. A merged state equation is expressed as

(45) $$\begin{array}{c} x\left(t\right)=x_{k}+n\;\;D+\eta \left(t-t_{k}\right)+\sigma B\left(t-t_{k}\right). \end{array}$$

Equation (45) is a variant of the Wiener process. According to the Wiener process property and the definition determined by equation (43), the RUL probability density function conditioned on *n* Poisson shocks, *x*_*k*_, ***θ***, and **Y**_*k*_ obeys the inverse Gaussian distribution, i.e.,

(46) $$\begin{array}{c} \left(\left.T_{k}\right|n,x_{k},\theta ,Y_{k}\right)∼IG\left(\frac{\left(w-x_{k}-nD\right)}{\eta },\frac{{\left(w-x_{k}-nD\right)}^{2}}{\sigma^{2}}\right), \end{array}$$

where *T*_*k*_ represents the RUL at time *k*. Its mean is given by *E*(*T*_*k*_|*n*, *x*_*k*_, ***θ***, **Y**_*k*_)=(*w* − *x*_*k*_ − *n*  *D*)/*η* and its PDF is formulated by

(47) $$\begin{array}{c} f\left(\left.T_{k}\right|n,x_{k},\theta ,Y_{k}\right)=\frac{w-x_{k}-nD}{\sqrt{2\pi T_{k}^{3}\sigma^{2}}}\exp\left(-\frac{{\left(w-\eta T_{k}-x_{k}-nD\right)}^{2}}{2\sigma^{2}T_{k}}\right). \end{array}$$

Equation (47) does not take into account the simulated degradation state distribution obtained by the parallel simulation system. The distribution reflects the uncertainty of the simulated degradation state. Integrating it into the RUL distribution determined by equation (47) can improve the prediction accuracy and enhance the prediction rationality. However, it will involve complex integral operation. So a lemma is utilized to obtain the probability density function of the RUL.

**Lemma 1 .**

Supposing that *Ω* ~ N(*γ* − *ξ* ^2^) and *A*, *B*, and *C* are all constant,

(48) $$\begin{array}{c} E_{\Omega }\left(\left(A-\Omega \right)\exp\left(-\frac{{\left(B-\Omega \right)}^{2}}{2C}\right)\right)=\sqrt{\frac{C}{\xi^{2}+C}}\left(A-\frac{\xi^{2}B+\gamma C}{\xi^{2}+C}\right)\exp\left(-\frac{{\left(B-\gamma \right)}^{2}}{2\left(\xi^{2}+C\right)}\right). \end{array}$$

The proof of Lemma 1 can refer to literature [48]. On the basis of Lemma 1, a theorem is proposed to achieve the RUL distribution.

**Theorem 1 .**

For the hybrid degradation process {*x*(*t*), *t* ≥ 0}, the RUL distribution at time *k* can be given by

(49) $$\begin{array}{c} f_{T}\left(\left.T_{k}\right|\theta ,Y_{k}\right)=\overset{+\infty }{\underset{n=0}{\sum }}\left\{\frac{{\left(\rho T_{k}\right)}^{n}\left[\sigma^{2}\left(w-{\hat{x}}_{k\left|k\right.}-nD\right)+\eta P_{k\left|k\right.}\right]}{\sqrt{2\pi {\left(P_{k\left|k\right.}+\sigma^{2}T_{k}\right)}^{3}}\cdot n!}\cdot \exp\left(-\rho T_{k}-\frac{{\left(w-\eta T_{k}-{\hat{x}}_{k\left|k\right.}-nD\right)}^{2}}{2\left(P_{k\left|k\right.}+\sigma^{2}T_{k}\right)}\right)\right\}. \end{array}$$

According to the form of the specific HWSSM and IMM filtering algorithm, *x*_*k*_ follows the normal distribution, i.e., $x_{k}∼N\left({\hat{x}}_{k\left|k\right.},P_{k\left|k\right.}\right)$. Let *p*(*x*_*k*_|**Y**_*k*_) express the conditional PDF about **Y**_*k*_ of *x*_*k*_. Based on the total probability theorem, the simulated degradation state distribution is integrated into the inverse Gaussian distribution and gives

(50) $$\begin{array}{c} f_{T}\left(\left.T_{k}\right|\theta ,Y_{k}\right)=\int_{-\infty }^{+\infty }f_{T}\left(\left.T_{k}\right|x_{k},\theta ,Y_{k}\right)p\left(\left.x_{k}\right|Y_{k}\right)dx_{k}=\overset{+\infty }{\underset{n=0}{\sum }}\int_{-\infty }^{+\infty }f_{T}\left(\left.T_{k}\right|n,x_{k},\theta ,Y_{k}\right)p\left(\left.x_{k}\right|Y_{k}\right)dx_{k}p\left(n\right)\\ =\overset{+\infty }{\underset{n=0}{\sum }}E_{x_{k}}\left[f_{T}\left(\left.T_{k}\right|n,x_{k},\theta ,Y_{k}\right)\right]p\left(n\right). \end{array}$$

According to Lemma 1, let *A*=*w* − *n*  *D*, *B*=*w* − *ηT*_*k*_ − *n*  *D*, *C*=*σ* ^2^ *T*_*k*_, *Ω*=*x*_*k*_, $\gamma ={\hat{x}}_{k\left|k\right.}$, and *ξ* ^2^=*P*_*k*|*k*_ and calculate the mathematical expectation about *x*_*k*_ of *f*(*T*_*k*_|*x*_*k*_, ***θ***, **Y**_*k*_). Then, the weighted sum with the occurrence probability of the *n* Poisson shocks is calculated. As a result, equation (49) is obtained. This completes the proof of Theorem 1.

According to the property of the mathematical expectation and equation (49), the expected value of the RUL can be obtained by

(51) $$\begin{array}{c} E\left(\left.T_{k}\right|\theta ,Y_{k}\right)=E_{x_{k}\left|\theta \right.,Y_{k}}\left[E_{T}\left(\left.T_{k}\right|x_{k},\theta ,Y_{k}\right)\right]=E_{x_{k}\left|\theta \right.,Y_{k}}\left\{E_{T}\left[\overset{+\infty }{\underset{n=0}{\sum }}f_{T}\left(\left.T_{k}\right|n,x_{k},\theta ,Y_{k}\right)p\left(n\right)\right]\right\}. \end{array}$$

Obviously, it is difficult to directly obtain the analytical expression of RUL's mathematical expectation by using the above equation. Therefore, according to the definition of mathematical expectation, the parallel simulation system uses numerical integration to calculate the mathematical expectation value of the RUL, i.e.,

(52) $$\begin{array}{c} E_{T}\left(T_{k}\right)=\int_{0}^{+\infty }T_{k}\cdot f_{T}\left(\left.T_{k}\right|\theta ,Y_{k}\right)dT_{k}. \end{array}$$

According to the equations (49) and (52), the parallel simulation system can support maintenance decision-making by calculating the probability density function of the RUL, and its expected value in an online and real-time manner.

## 5. A Case Study

### 5.1. Data Introduction

**Figure 2:**
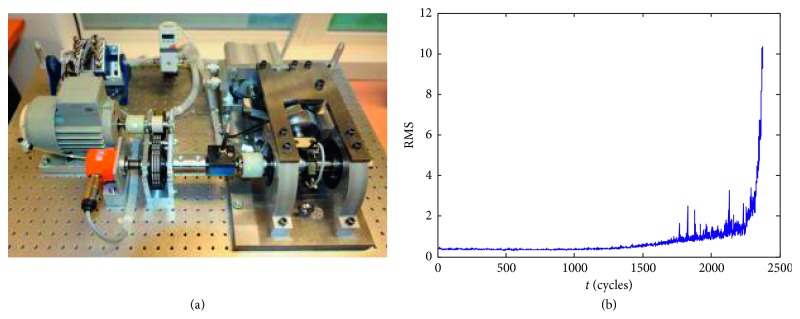
Test platform and degradation data. (a) PRONOSTIA platform. (b) RMS of bearing 1–3.

The performance degradation of bearings of mechanical equipment is a typical hybrid degradation process with shock characteristic. This paper uses the life test data of a bearing of the IEEE PHM 2012 Prediction Competition [49] to verify the parallel simulation method considering model soft switch. These data are provided by the FEMTO-ST Institute in France. The life test is conducted on the PRONOSTIA platform shown in Figure 2(a), and the test data have been widely used in method validation in the reliability field in recent years. The life test is divided into 3 different working conditions, while the first working condition is rotating speed of 1800 rpm with a load of 4000 N. The record time of the test data is from 2010/11/17 08:33:01 to 2010/11/17 15:08:41, and the sampling frequency of the vibration signal is 25.6 kHz with the sampling interval of 10 s. A total of 2375 data samples are collected in the first working condition. In the case study, the life test data of the third bearing under the first working condition are used to validate the method. Particularly, the bearing is called bearing 1–3.

The root mean square (RMS) value of the vibration signal is a commonly used degradation feature and it is calculated by

(53) $$\begin{array}{c} \text{RMS}=\sqrt{\frac{1}{N}\overset{N}{\underset{i=1}{\sum }}e_{i}^{2}}, \end{array}$$

where *N* is the sampling point number and satisfies *N*=2560 and *e*_*i*_ denotes the vibration acceleration signal at the *i*th sampling point. The RMS of bearing 1–3 is shown in Figure 2(b). It can be seen that the shock characteristic of the performance degradation process for bearing 1–3 is obvious, indicating that the data are suitable for verifying the method. The RMS begins to change significantly after the 1500th monitoring time, which is regarded as the starting time of RUL prediction oriented parallel simulation. The failure criterion of the bearing is that the vibration intensity of the original signal reached 20 g at the 2341th monitoring time, and the corresponding RMS value is 4.7145. As a result, the failure threshold is set to the RMS value at the 2341th monitoring time, i.e., *w*=4.7145.

### 5.2. Model Evolution and RUL Real-Time Prediction

**Figure 3:**
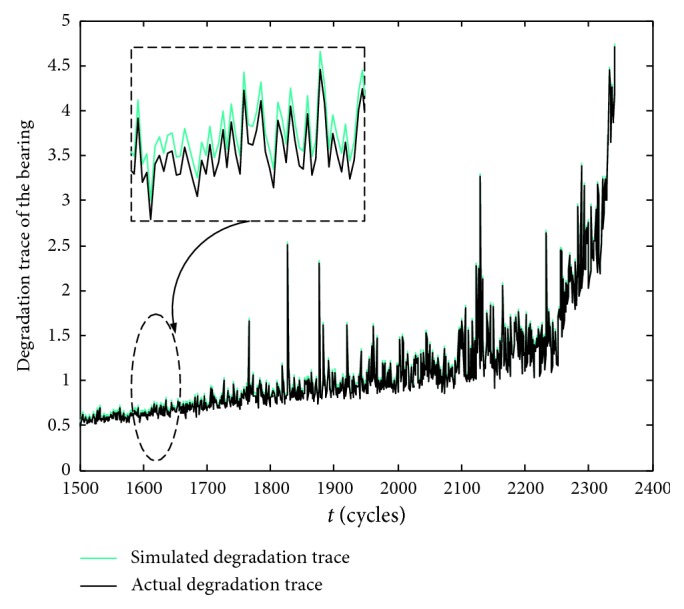
Comparison of the degraded traces.

The initial simulation configurations include *x*_0_=0.2, *η*=0.02, *σ*=0.5, *D*=0.02, *ρ*=0.5, *τ*=1, and *ϕ*=0.1. Furthermore, the vector of initial model probability is *μ*_0_=[0.6  0.4]^T^, i.e., *μ*_0_^1^=0.6 and *μ*_0_^2^=0.4. The model transition probability matrix is chosen as *P*=[0.5 0.5; 0.6 0.4]. The comparison of the degraded trajectories is shown in Figure 3. It shows that the difference between the simulated degradation trajectory and the actual degradation trajectory is minimal, indicating that the simulation output can effectively approach the actual degradation process driven by the real-time degraded data. In order to quantify the comparison results, the root mean square error (RMSE) is given by

(54) $$\begin{array}{c} \text{RMSE}=\sqrt{\frac{1}{r}\overset{r}{\underset{k=1}{\sum }}{\left(\frac{y_{k}-{\hat{x}}_{k\left|k\right.}}{y_{k}}\right)}^{2}}\times 100\%, \end{array}$$

where *r*(*r*=842) denotes the number of monitoring points. After calculation, the RMSE of the simulated degradation trajectory and the actual observed degradation trajectory is only 3.497%, which fully demonstrates that the parallel simulation considering model soft switch can effectively model and simulate the performance degradation process with discrete shocks of bearing 1–3.

**Figure 4:**
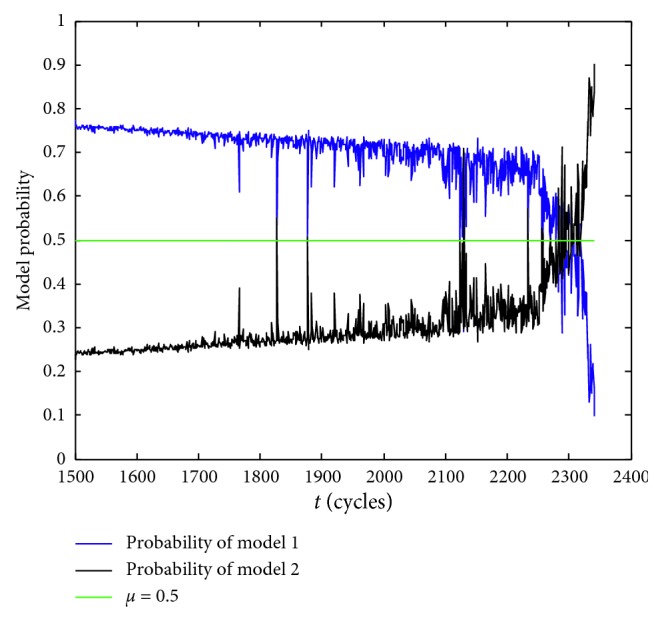
Model probability.

The model probability is shown in Figure 4. The Poisson shocks characteristic is not significant in the monitoring period from *t*_1500_ to *t*_1765_, and the linear degradation characteristic is more obvious. The probability of the linear degradation model which is noted as Model 1 is obviously higher than that of the degradation model with discrete Poisson shocks which is noted as Model 2. The model probabilities of the two models are about 0.74 and 0.26, respectively. In this monitoring period, the dominant model is Model 1. However, as time passes, the Poisson shock characteristic becomes more and more prominent, especially at the moments *t*_1766_, *t*_1827_, *t*_1877_, *t*_2130_, *t*_2234_, etc. The probability of Model 2 generally shows a dynamic upward trend. Conversely, the probability of Model 1 shows a dynamic decline trend. In the late degradation stage, the probability of Model 2 surpasses the probability of Model 1, which shows that the former model is more suitable for describing the current degradation process. Above all, the parallel simulation method considering model soft switch can effectively meet the needs of model suitability for RUL prediction. It is worth noting that the probability curves of the two models are symmetric about the probability *μ*=0.5.

**Figure 5:**
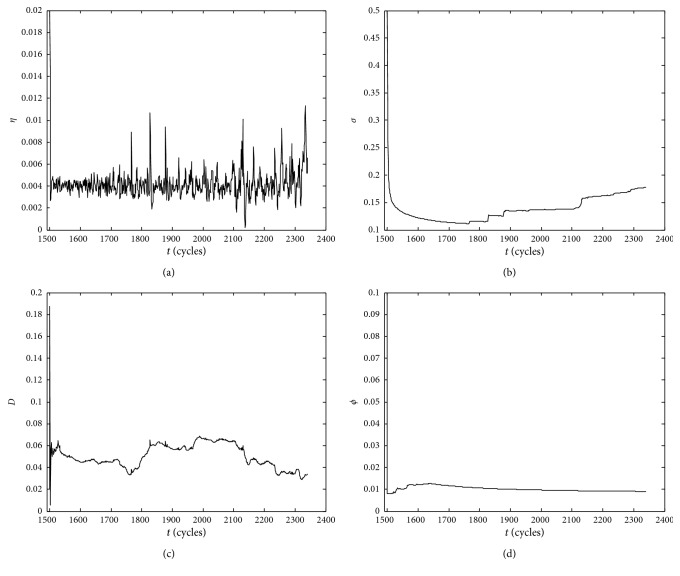
Parameters evolution of HWSSM. (a) Parameter *η* evolution. (b) Parameter *σ* evolution. (c) Parameter *D* evolution. (d) Parameter *ϕ* evolution.

With the dynamic injection of the observed degradation data, the parallel simulation system uses the IMM-EM algorithm to perform model evolution. The estimated results of model parameters are shown in Figure 5. It shows that the drift coefficient *η* fluctuates around 0.004 with the fluctuation range [0, 0.012]. In addition, the fluctuations are larger at the monitoring times *t*_1766_, *t*_1827_, etc., reflecting the accelerated degradation rate of bearings 1–3. The diffusion coefficient *σ* can converge quickly. When the shocks characteristic is obvious, *σ* fluctuates greatly and reaches a new convergence state, which is conducive to obtaining a stable remaining life probability density function. Furthermore, the convergence value of *σ* is rather small, which is helpful to obtain a narrower PDF of the RUL and improve the accuracy of RUL prediction. The damage *D* caused by Poisson shocks fluctuates dynamically within the interval [0.03, 0.07]. Considering the damage into the RUL prediction, it can effectively reduce the occurrence of “lack maintenance.” Parameter *ϕ* converges faster and the convergence value is about 0.01, reflecting that the fluctuation of measurement error is gradually stable.

**Figure 6:**
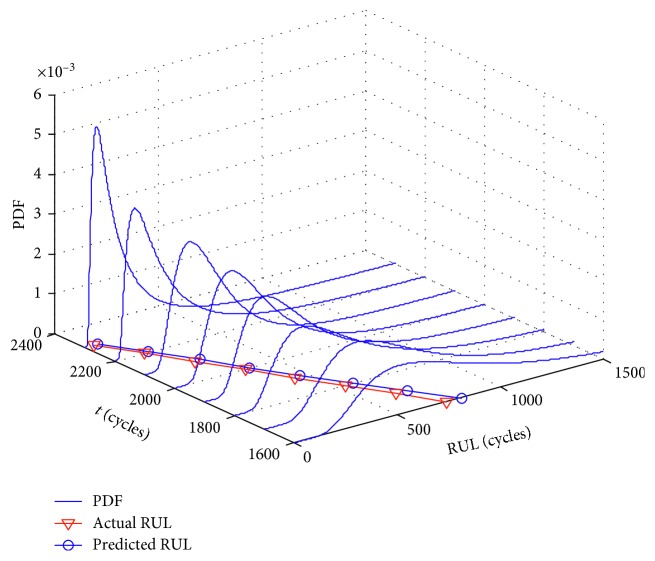
PDF of the RUL for bearing 1–3 at different monitoring time.

With the execution of model soft switch and parameters evolution, the parallel simulation system uses equations (49) and (52) to predict the RUL of bearings 1–3, including the PDF of the RUL and its mathematical expectation.  Figure 6 shows the PDF curves predicted at eight different monitoring times from *t*_1600_ to *t*_2300_ with the prediction interval of 100 monitoring points.

According to Figure 6, at each monitoring time of predicting the RUL, the PDF curve of the RUL can effectively cover the actual RUL. As the degradation data of bearing 1–3 continuously accumulates, the PDF curve of the RUL gradually narrows with the weaker “right-biased” characteristic and the stronger “normal” characteristic. The prognostic results indicate that the model matching degree is gradually improved, and the model parameters are more accurate. As a result, the uncertainty of the RUL prediction is getting smaller. This is due to the model soft switch and parameters online estimation. In addition, the error between the mathematical expectation of the RUL and the actual RUL is small. And also, the mathematical expectation of the RUL is close to the peak of the PDF curve, indicating that the uncertainty of the PDF is small, and the prognostic results can provide an important basis for maintenance decision.

### 5.3. Comparative Study

**Figure 7:**
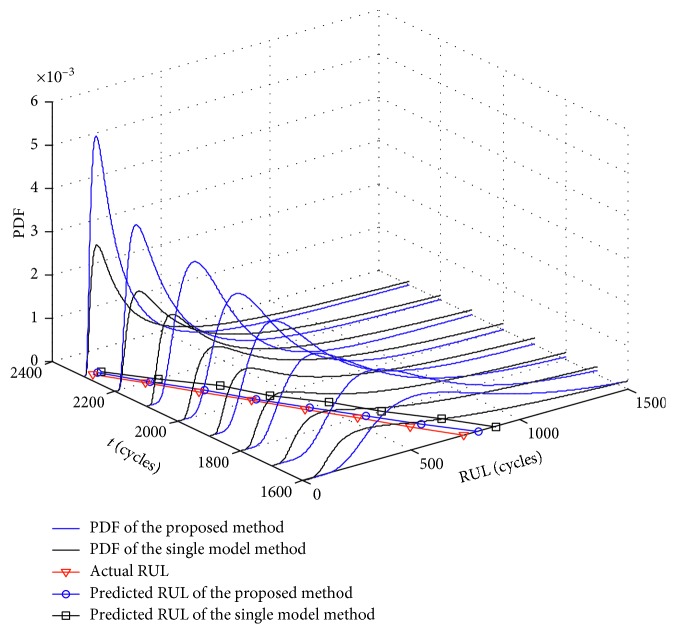
Comparative results of the RUL prediction.

To further verify the validity of the parallel simulation method considering the model soft switch, the comparative study is performed by comparing with the method without considering the model soft switch which can be found in [50]. In that case, the single model is used to only execute model parameters evolution, i.e., the state equation equation (2) and the observation equation (6). So the method is called the single-model method. The equations of model parameters evolution and RUL prediction for the single-model method are given in Appendix. The RUL prognostic results of the single-model method and the proposed method are shown in Figure 7.

**Figure 8:**
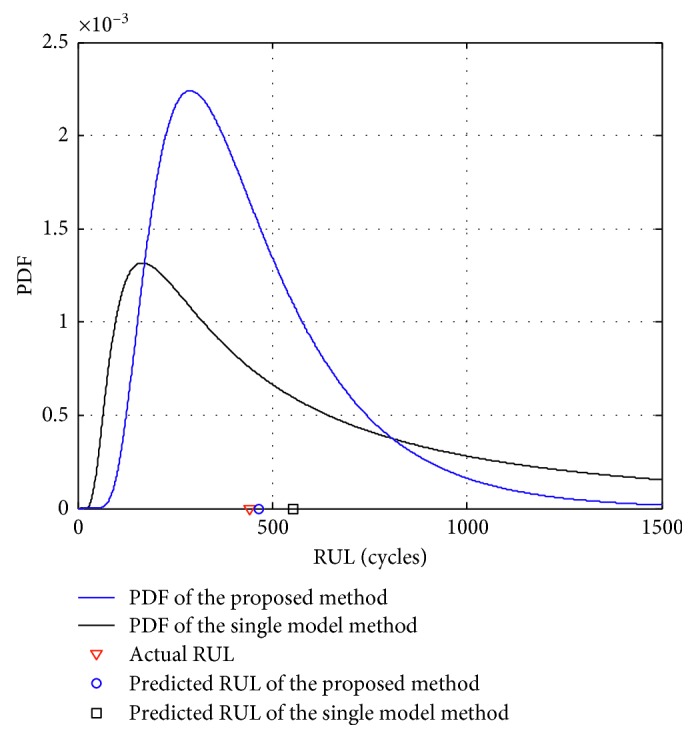
Comparative results of the RUL prediction at the 1900th monitoring time.

The PDF curves of the single-model method are flatter than that of the proposed method, which indicates that it has stronger uncertainty. Moreover, the “right-biased” characteristic of the PDF curves of the single-model method is more obvious and there is a long “tail.” Although the PDF curves of the single-model method can also cover the true RUL, the flat distribution of the RUL and the obvious “right-biased” characteristic cause the prognostic results to be unfavorable to the maintenance decision. In addition, at each monitoring time of predicting the RUL, the mathematical expectation of the RUL obtained by the single-model method is all greater than that of the proposed method, implying the larger prediction error. On the contrary, the proposed method has better performance, and the prognostic results at the 1900th monitoring time are taken as examples for analysis. As shown in Figure 8, the PDF curves obtained by the proposed method is more compact with less uncertainty, and the corresponding peak value is 2.24 × 10^−3^, which is bigger than the peak value 1.31 × 10^−3^ achieved by the single-model method. Besides, the RUL corresponding to the peak value of the proposed method and the single-model method are 292 cycles and 170 cycles, respectively. Considering that the true RUL at the 1900th monitoring time is 441 cycles, it shows that the RUL corresponding to the peak value of the proposed method is closer to the true RUL than that of the single-model method. Additionally, the mathematical expectation of the RUL obtained by the proposed method and the single-model method are 465.92 cycles and 553.67 cycles, respectively, also illustrating that the prediction error obtained by the proposed method is smaller than that of the single-model method.

In order to further quantify the comparison results of the RUL prediction, two quantitative indicators, the average relative accuracy and the total mean square error (TMSE), are introduced. At the monitoring time *k*, the definition of the relative prediction accuracy of the RUL is given by

(55) $$\begin{array}{c} {\text{RA}}_{k}=1-\frac{\left|{\tilde{T}}_{k}-T_{k}\right|}{{\tilde{T}}_{k}}, \end{array}$$

where ${\tilde{T}}_{k}$ denotes the true RUL at time *k*. On the basis of RA_*k*_, the average relative accuracy MRA is defined as

(56) $$\begin{array}{c} \text{MRA}=\frac{1}{p}\overset{p}{\underset{k=1}{\sum }}\text{R}{\text{A}}_{k}, \end{array}$$

where *p* represents the number of monitoring time points used to predict the RUL. According to the definition of MRA, it satisfies MRA ∈ [0,1] and the bigger MRA indicates the higher prediction accuracy with respect to the RUL. In addition, the TMSE between the actual RUL and the mathematical expectation of the predicted RUL is defined as

(57) $$\begin{array}{c} \text{TMSE}=\overset{p}{\underset{k=1}{\sum }}E\left[{\left(T_{k}-{\tilde{T}}_{k}\right)}^{2}\right]=\overset{p}{\underset{k=1}{\sum }}\int_{0}^{\infty }{\left(T_{k}-{\tilde{T}}_{k}\right)}^{2}f_{T}\left(\left.T_{k}\right|\theta ,Y_{k}\right)dT_{k}. \end{array}$$

Based on the definition of the TMSE, the smaller TMSE implies the more accurate prediction result. The calculated comparison results are shown in Table 1. According to Table 1, the MRA obtained by the proposed method is obviously larger than that of the single-model method, indicating the higher relative prediction accuracy. The TMSE of the proposed method is much smaller than that of the single-model method, implying the smaller RUL prediction error.

## 6. Conclusions and Future Perspectives

Equipment parallel simulation is an emerging simulation paradigm, which has an important concept of model evolution. Based on the background of RUL prediction for the hybrid degradation equipment with continuous degradation and discrete shocks, the hybrid degradation equipment RUL prediction oriented parallel simulation considering model soft switch is studied, including parallel simulation modeling, model evolution, RUL prediction, and case study. Under the modeling framework of SSM, two different models are constructed by using the Wiener process and the effect of Poisson shocks. One model describes a continuous degradation process, and the other model expresses a degradation process with discrete shocks. Owing to the discrete, unknown characteristics of the shocks, it is not possible to directly determine the model morphology at a specific time. Therefore, the forward IMM filtering is utilized to dynamically calculate the probabilities of different models, achieving the model soft switch. Then, the model probability-based weighted summation is performed to obtain the simulated degradation state estimation. On the basis of model soft switch, the evolution of model parameters based on the EM algorithm is studied. Through the iteration between model soft switch and model parameters evolution, the output of the simulation model can dynamically approach the actual degradation process. In order to realize RUL real-time prediction, the unknown Poisson shocks is firstly integrated into the Wiener process. According to the concept of the first hitting time and the mathematical property of the Wiener process, the RUL distribution which neglects the simulated degradation state distribution is achieved and subjects to the inverse Gaussian distribution. Then, based on the total probability theorem, the simulated degradation state distribution is integrated into the inverse Gaussian distribution to obtain the analytical expression of the PDF of the RUL. A bearing degradation data with typical hybrid degradation characteristic is regarded as the data-driven source to verify the proposed method considering model soft switch. The results show that the proposed method can effectively model the bearing performance degradation process. Comparative study shows that the PDF of the RUL acquired by the proposed method has a less uncertainty and higher prediction accuracy than that of the single-model method. This paper researches and perfects the modeling method, model evolution mechanism, and RUL prediction method of parallel simulation in the field of equipment RUL prediction, which is helpful to promote the application of parallel simulation in actual equipment maintenance support.

There are several underlying directions deserving further implementation. First, this paper only considers the hybrid degradation with linear continuous degradation and discrete shocks. It is a challenging work further considering the hybrid degradation with nonlinear continuous degradation and discrete shocks. The mechanisms of model soft switch and parameters evolution will be more complicated and difficult. Additionally, the multistage case can be considered, and the corresponding model dynamically evolution is deserved to research.

## Figures and Tables

**Table 1:** Comparative results of RUL prediction.

Methods	Indicators	
	MRA	TMSE (×10^4^)
Single-model method	0.6427	13.5037
Proposed method	0.8607	5.4611

## Data Availability

The bearing degradation data supporting the findings of this study are from previously reported studies and datasets, which have been cited. The processed data are available at “http://www.femto-st.fr/en/Researchdepartments/AS2M/Research-groups/PHM/IEEE-PHM-2012-Data-challenge” or in “P. Nectoux, R. Gouriveau, K. Medjaher, E. Ramasso, B. Morello, N. Zerhouni, C. Varnier, “PRONOSTIA: an experimental platform for bearings accelerated life test,” IEEE International Conference on Prognostics and Health Management, Denver, CO, USA, 2012.”

## Appendix

According to the single-model method, it can be known that *x*_*i*_|*x*_*i*−1_ ~ *N*(*x*_*i*−1_+*ητ*_*i*_, *σ* ^2^ *τ*_*i*_) and *y*_*i*_|*x*_*i*_ ~ *N*(*x*_*i*_, *ϕ* ^2^). The joint log-likelihood function *L*(**X**_*k*_, **Y**_*k*_|***θ***) can be formulated as

(A.1) $$\begin{array}{c} L\left(X_{k},\left.Y_{k}\right|\theta \right)=\ln\;\;p\left(X_{k},\left.Y_{k}\right|\theta \right)\\ =\ln\left(p\left(\left.x_{0}\right|\theta \right)\overset{k}{\underset{i=1}{\prod }}p\left(\left.x_{i}\right|x_{i-1},\theta \right)\overset{k}{\underset{i=1}{\prod }}p\left(\left.y_{i}\right|x_{i},\theta \right)\right)\\ =-\frac{k+1}{2}\ln\;\;2\pi -\frac{1}{2}\ln\;\;\Sigma_{0}-\frac{{\left(x_{0}-\mu_{0}\right)}^{2}}{2\Sigma_{0}}-\frac{1}{2}\overset{k}{\underset{i=1}{\sum }}\ln\;\;\sigma^{2}\tau_{i}-\frac{1}{2\sigma^{2}}\overset{k}{\underset{i=1}{\sum }}\left(\frac{{\left(x_{i}-x_{i-1}-\eta \tau_{i}\right)}^{2}}{\tau_{i}}\right)-\frac{k}{2}\ln\;\;\phi^{2}-\frac{1}{2\phi^{2}}\overset{k}{\underset{i=1}{\sum }}{\left(y_{i}-x_{i}\right)}^{2}. \end{array}$$

Based on the Bayesian theorem and the Markov property and omitting independent parts, *Q*(***θ***, ***θ***_*k*_^(*j*)^) can be expanded as

(A.2) $$\begin{array}{c} Q\left(\theta ,\theta_{k}^{\left(j\right)}\right)\propto -\frac{1}{2}\ln\;\;\Sigma_{0}-\frac{{\hat{x}}_{0\left|k\right.}^{2}+P_{0\left|k\right.}+\mu_{0}^{2}-2\mu_{0}{\hat{x}}_{0\left|k\right.}}{2\Sigma_{0}}-\frac{1}{2}\overset{k}{\underset{i=1}{\sum }}\ln\;\;\sigma^{2}\tau_{i}-\frac{1}{2\sigma^{2}}\overset{k}{\underset{i=1}{\sum }}\left(\frac{\Gamma_{i}-2\eta \tau_{i}{ϒ}_{i}+{\left(\eta \tau_{i}\right)}^{2}}{\tau_{i}}\right)-\frac{k}{2}\ln\;\;\phi^{2}-\frac{1}{2\phi^{2}}\overset{k}{\underset{i=1}{\sum }}\left(y_{i}^{2}+P_{i\left|k\right.}+{\hat{x}}_{i\left|k\right.}^{2}-2y_{i}{\hat{x}}_{i\left|k\right.}\right), \end{array}$$

where ${ϒ}_{i}={\hat{x}}_{i\left|k\right.}-{\hat{x}}_{i-1\left|k\right.}$.

Through the derivation of the M-step, the parameters evolution of the single-model method at the *j*th iterative step is determined by

(A.3) $$\begin{array}{c} \left\{\begin{array}{c} {\hat{\mu }}_{0}={\hat{x}}_{0\left|k\right.},\\ {\hat{\Sigma }}_{0}=P_{0\left|k\right.},\\ \hat{\eta }=\frac{\sum_{i=1}^{k}{ϒ}_{i}}{\sum_{i=1}^{k}\tau_{i}},\\ {\hat{\sigma }}^{2}=\frac{1}{k}\overset{k}{\underset{i=1}{\sum }}\left(\Gamma_{i}-2\eta \tau_{i}{ϒ}_{i}+{\left(\eta \tau_{i}\right)}^{2}\right),\\ {\hat{\phi }}^{2}=\frac{1}{k}\overset{k}{\underset{i=1}{\sum }}\left(y_{i}^{2}+P_{i\left|k\right.}+{\hat{x}}_{i\left|k\right.}^{2}-2y_{i}{\hat{x}}_{i\left|k\right.}\right). \end{array}\right. \end{array}$$

Via the iteration of Kalman filtering and EM algorithm, the model parameters can be obtained by equation (A.3). Then, the RUL distribution at time *k* of the single-model method can be calculated by

(A.4) $$\begin{array}{c} f\left(\left.T_{k}\right|\theta ,Y_{k}\right)=\int_{-\infty }^{+\infty }f\left(\left.T_{k}\right|x_{k},\theta ,Y_{k}\right)p\left(\left.x_{k}\right|Y_{k}\right)dx_{k}\\ =\frac{1}{\sqrt{2\pi T_{k}^{2}\left(P_{k\left|k\right.}+\sigma^{2}T_{k}\right)}}\left(w-\frac{P_{k\left|k\right.}\left(w-\eta T_{k}\right)+{\hat{x}}_{k\left|k\right.}\sigma^{2}T_{k}}{P_{k\left|k\right.}+\sigma^{2}T_{k}}\right)\exp\left(-\frac{{\left(w-\eta T_{k}-{\hat{x}}_{k\left|k\right.}\right)}^{2}}{2\left(P_{k\left|k\right.}+\sigma^{2}T_{k}\right)}\right). \end{array}$$

The proof of equation (A.4) is similar to Theorem 1. The RUL expected value can be formulated as follows:

(A.5) $$\begin{array}{c} E\left(\left.T_{k}\right|\theta ,Y_{k}\right)=E_{x_{k}\left|\theta \right.,Y_{k}}\left[E\left(\left.T_{k}\right|x_{k},\theta ,Y_{k}\right)\right]=E_{x_{k}\left|\theta \right.,Y_{k}}\left[\frac{w-x_{k}}{\eta }\right]=\frac{w-{\hat{x}}_{k\left|k\right.}}{\eta }. \end{array}$$
